# The role of drug resistance in poor viral suppression in rural South Africa: findings from a population-based study

**DOI:** 10.1186/s12879-020-4933-z

**Published:** 2020-03-26

**Authors:** Sheri A. Lippman, Alyssa C. Mooney, Adrian Puren, Gillian Hunt, Jessica S. Grignon, Lisa M. Prach, Hailey J. Gilmore, Hong-Ha M. Truong, Scott Barnhart, Teri Liegler

**Affiliations:** 1 Division of Prevention Science, Department of Medicine, University of California San Francisco, 550 16th Street, 3rd Floor, San Francisco, CA 94158-2549 USA; 2 Department of Epidemiology, University of California San Francisco, San Francisco, USA; 3 National Institute for Communicable Diseases/NHLS, Johannesburg, South Africa; 4 Division of Virology, School of Pathology, University of the Witwatersrand, Johannesburg, South Africa; 5 Department of Global Health, University of Washington, Seattle, USA; 6 International Training and Education Center for Health (I-TECH) South Africa, Pretoria, Republic of South Africa; 7 Department of Medicine, University of California San Francisco, San Francisco, USA

**Keywords:** South Africa, HIV drug resistance, Surveillance, viral suppression, Virological failure, Antiretroviral therapy (ART), ART adherence

## Abstract

**Background:**

Understanding factors driving virological failure, including the contribution of HIV drug resistance mutations (DRM), is critical to ensuring HIV treatment remains effective. We examine the contribution of drug resistance mutations for low viral suppression in HIV-positive participants in a population-based sero-prevalence survey in rural South Africa.

**Methods:**

We conducted HIV drug resistance genotyping and ART analyte testing on dried blood spots (DBS) from HIV-positive adults participating in a 2014 survey in North West Province. Among those with virologic failure (> 5000 copies/mL), we describe frequency of DRM to protease inhibitors (PI), nucleoside reverse transcriptase inhibitors (NRTI), and non-nucleoside reverse transcriptase inhibitors (NNRTI), report association of resistance with antiretroviral therapy (ART) status, and assess resistance to first and second line therapy. Analyses are weighted to account for sampling design.

**Results:**

Overall 170 DBS samples were assayed for viral load and ART analytes; 78.4% of men and 50.0% of women had evidence of virologic failure and were assessed for drug resistance, with successful sequencing of 76/107 samples. We found ≥1 DRM in 22% of participants; 47% were from samples with detectable analyte (efavirenz, nevirapine or lopinavir). Of those with DRM and detectable analyte, 60% showed high–level resistance and reduced predicted virologic response to ≥1 NRTI/NNRTI typically used in first and second-line regimens.

**Conclusions:**

DRM and predicted reduced susceptibility to first and second-line regimens were common among adults with ART exposure in a rural South African population-based sample. Results underscore the importance of ongoing virologic monitoring, regimen optimization and adherence counseling to optimize durable virologic suppression.

## Background

The UNAIDS HIV epidemic targets for detection, sustained antiretroviral therapy (ART), and viral suppression propose that overall community viral suppression should reach 73% by 2020 [1]. With testing and treatment increasingly available and universal in sub-Saharan Africa, home to the majority of HIV cases and greatest need for treatment scale up [2], meeting this goal should be attainable, particularly as ART has increasingly reduced morbidity and mortality and has been demonstrated to substantially reduce further transmission [3–6]. To reap the benefits of ART and achieve widespread viral suppression, however, both access to and consistent adherence to medication is critical for achieving durable viral suppression and preventing drug resistance [3, 7–10].

Among patients in clinical research cohorts in sub-Saharan Africa with access to virologic monitoring, viral suppression at 12 months has been estimated between 84 and 90% among those on ART [11–13]. In South Africa, findings of the National HIV Prevalence, Incidence, Behaviour and Communication Survey 2017 similarly estimated that 89.9 and 82.1% of females and males on ART were virally suppressed [14]. Less is known about prevalence of viral suppression in the general population, particularly in rural areas, where monitoring is less consistent [15]. One population-based study covering 32 rural Kenyan and Ugandan communities noted that 45% of HIV-positive individuals had evidence of viral suppression prior to intensive interventions to improve ART initiation [16]. The recent Universal Test and Treat trial in Kwa Zulu Natal similarly noted high viral suppression rates for those on ART (85%), but an overall suppression rate of 49% among all PLHIV in 2016 [17], lower than the 2016–2017 PopART trial estimates of 63–72% virally suppressed in community cohorts [18].

While known factors contribute to virological failure (intermittent adherence to medication resulting in non-suppressive drug levels in a setting of ongoing viral replication, transmitted resistance), it remains unknown what proportion of those undergoing virological failure can be attributed to each factor. Studies in sub-Saharan Africa have found drug resistance mutations in 6–14% of ART-naïve patients [11, 19, 20], and 84–89% of those with virological failure who initiated ART ≥12 months prior [19, 21]. Results from a systematic review and meta-analysis estimated a prevalence of pretreatment non-nucleotide reverse-transcriptase inhibitor (NNRTI) resistance of 11% in southern Africa [22]. Results from one of few population-based studies in South Africa indicate that transmitted resistance is increasing; from 0% in 2010, to 5 and 7% in 2011 and 2012, respectively [23]. Understanding factors driving virological failure, including the contribution of both pre-treatment drug resistance and acquired resistance, is critical to ensuring treatment remains effective and that existing first-line regimens can be preserved. Data are scarce on the prevalence of genotypic resistance in rural areas of Sub-Saharan Africa [15] and rarely is genotypic resistance data available from population-based sampling, coincident with both reported adherence and known ART exposure.

We conducted a population-based bio-behavioral survey in 2014 to characterize the HIV care continuum in a rural district of North West Province, an area of the country with substantial burden of disease and little available data [24]. We noted that while > 90% of men and women in care reported taking ART, only an estimated 29% of men and 60% of women in care achieved virologic suppression (< 5000 copies/mL) measured from dried blood spots (DBS). To understand the discrepancy between reported ART intake and viral suppression and assess contributing factors to the low suppression rates, we assessed all HIV-positive participants for antiretroviral drug exposure and performed drug resistance testing among those not suppressed. In this manuscript we examine the potential contribution of drug resistance mutations for low viral suppression in this HIV-positive, rural South African, population-based sample, discussing implications for future programming.

## Methods

### Study setting

Data were collected from January–March 2014 in Lekwa-Teemane and Greater Taung sub-districts, within Dr. Ruth Segomotsi Mompati (RSM) District of North West Province, South Africa. RSM is comprised of both rural and peri-urban areas, with an economy centered on beef production and agriculture. The study area includes approximately 230,000 people, the majority of whom speak Setswana. North West Province has the fourth highest HIV prevalence in South Africa, with an estimated prevalence of 20.3% in the adult population 15–49 years [25] and 29.2% in the antenatal population [26]. One quarter of households in North West are food insecure, reporting lack of money to buy food in the past year [27].

### Data collection

The 2014 bio-behavioral survey employed multi-stage cluster sampling, with twenty-three enumeration areas (EAs) selected proportionate to size in each sub-district by Statistics South Africa (StatsSA) using 2011 census data. In each EA up to 36 inhabited dwelling units (DUs) were randomly selected from the StatsSA sampling frame for inclusion in the sample and one adult (18–49 years) was randomly selected per DU for participation. Local, trained fieldworkers assessed eligibility criteria, including age (18–49), residence in the home, and ability to consent; obtained written informed consent; and conducted a survey by computer-assisted personal interviewing in a private location at the participant’s home. Survey questions included HIV testing history, known status, history of HIV care and treatment, ART initiation, and medication adherence. The fieldworkers then referred consenting participants for HIV rapid testing and DBS sample collection. Full data collection procedures have been described elsewhere [24].

For individuals consenting to HIV testing and sample collection, serostatus was determined using the Alere Determine HIV-1/2 rapid antibody test with finger-stick capillary blood (Alere Medical Co.,Ltd., Chiba, Japan) and, if reactive, confirmed using the First Response HIV 1–2.0 Rapid Whole Blood Test (Premier Medical Corporation Ltd., Daman, India). Participants with HIV-positive or discrepant results were asked to provide finger-prick blood for DBS using a Munktell filter card (Ahlstrom Munktell, Helsinki, Finland). Participants who declined HIV rapid testing in their home could provide blood for DBS for laboratory HIV diagnosis (serology: ELISA confirmed with Western blot).

### Laboratory methods

Viral Load: DBS cards were dried, stored under desiccant at ambient temperature, transported to the testing laboratory (Clinical Laboratory Services, Johannesburg) within six days of collection, and stored at − 70 °C. Viral load testing was performed using the COBAS AmpliPrep for sample preparation and COBAS TaqMan HIV-1 2.0 test (Roche Applied Science, Pleasanton CA, USA; lower limit of quantification 400 copies/mL) a previously validated method [28]. We opted to use a viral suppression cut-off of < 5000 copies/mL, a higher threshold than that recommended for plasma, as there is no definitive cut-point using DBS [29].

HIV Drug Resistance testing was performed using archived DBS with a VL > 5000 copies/mL at the National Institute for Communicable Diseases, Johannesburg, RSA. Spots were lysed in 2 ml of NucliSENS lysis buffer (Biomerieux, Germany) for 2 h at room temperature. Total nucleic acid was extracted using the NucliSENS EasyMAG® automated system according to the manufacturer’s instructions. Amplification of a 1084 bp polymerase chain reaction (PCR) fragment consisting of codons 1–99 of protease (Pro) and codons 1–250 of reverse transcriptase (RT) was performed as previously described [30]. Editing of sequences was performed using Recall software v2.10. Drug Resistance Mutations (DRM) and inferred susceptibility to individual ARV for RT and Pro regions were determined using the Stanford Drug Resistance Database V8.4 (http://hivdb.stanford.edu) [31].

ART Drug Exposure was determined by a validated qualitative liquid chromatography-tandem mass spectrometry (LC MS/MS) method for the determination of the presence or absence of various antiretroviral analytes against cutoff samples analysed at a known concentration (0.02 μg/ml). A method was validated for the qualitative determination of 9 antiretroviral drugs from DBS, and consisted of a protein precipitation, followed by high performance liquid chromatography with MS/MS detection using a gradient elution. Deuterated internal standards were used for each analyte. An AB Sciex API 4000 mass spectrometer at unit resolution in the multiple reaction monitoring (MRM) mode was used to monitor the transition of each of the protonated precursor ions. Analytes were assessed for the following three drugs: efavirenz (EFV), lopinavir (LPV), nevirapine (NVP), accounting for the majority of first and second line regimens, as tenofovir and emtricitabine are typically given in combination with efavirenz [32].

### Analysis

**Fig. 1:**
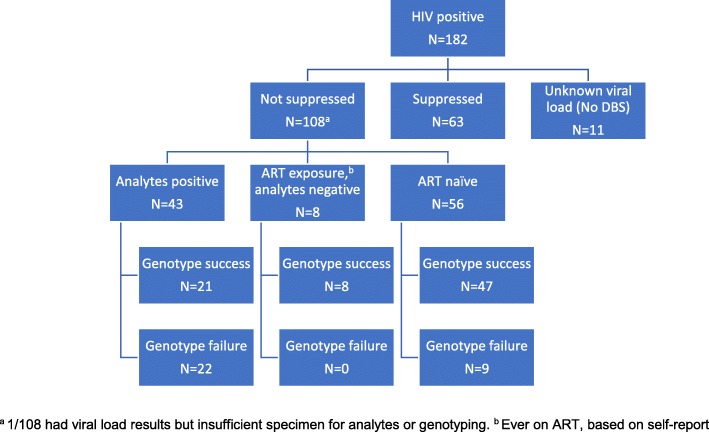
Participant viral suppression, medication history, and resistance status, North West Province, South Africa

Analyses aimed to characterize the scope of HIV drug resistance and types of DRM by treatment history among viremic HIV-positive participants. All analyses were weighted to account for sample design, with the exception of counts displayed in Fig. 1. Weights were created using the inverse probability of selection at each stage (EA, DU and person) and adjusted for non-response to reflect the municipality, age group and sex distributions within the target population [33]. Using the full survey population, we calculated weighted sample sizes, weighted proportions and 95% confidence intervals (CIs) to describe participant demographic characteristics and HIV-care indicators. We used Stata’s ‘subpop’ command to calculate estimates specific to HIV-positive participants, including full participant data in the calculation of standard errors. We used chi-square statistics to assess demographic differences between the HIV suppressed and non-HIV suppressed participants. When self-report and biomarkers were discrepant (i.e. participant stated not initiating ART but had positive analytes), we utilized the biomarker to indicate ART status.

Virological failure was defined as ≥5000 copies/mL measured from DBS, a conservative cut-off because of the potential for contributions of pro-viral DNA in the quantification using DBS instead of plasma RNA [29, 34]. We assessed presence of genotypic resistance to protease inhibitors (PI), NRTIs, and NNRTIs, and the association with ART status. ART status was categorized in three groups: those who had evidence of ART analytes, those who reported ART initiation but did not have evidence of analytes (likely inconsistent or interrupted treatment), and those who were ART-naïve, having reported never initiating ART and having no evidence of analytes.

Overall 71.7% of participants consented to HIV-testing (749/1044). Of participants with confirmed HIV-positive status, 94% provided DBS (171/182), however one DBS card had insufficient specimen for genotyping. To assess whether the population with missing DBS data differed from the population with complete data, we used multiple imputation to create 50 copies of the dataset with simulated values based on observed data; our analyses with imputed datasets produced adjusted estimates for uncertainty. Missing HIV and viral suppression status were imputed based on age, sex, education, food insecurity, and lifetime number of sexual partners. Imputation of missing viral load also incorporated ART initiation, adherence, and months since diagnosis. All analyses were performed using Stata 14 (StataCorp, College Station, TX, USA).

## Results

Among 182 seropositive participants confirmed by serial rapid testing or HIV DNA PCR, DBS were available for viral load and analyte testing from 170 participants (11 did not provide DBS and one had insufficient specimen for genotyping; Fig. 1). A total of 107 dB samples from individuals with viral load ≥5000 copies/ml were assayed for sequence-based drug resistance testing. While younger people were less likely to provide DBS, no differences were found in DBS provision by sex or education. Using imputed values for viral load and HIV status, viral suppression was not significantly different across DBS provision status.

### Participant characteristics

The final weighted sample reflects the sex and age distribution of the HIV-positive population of the sub-districts (Table 1). Overall, only 38.4% of the HIV-positive population had ≤5000 copies/mL detected (referred to in this manuscript as virally suppressed). Those who were suppressed were far more likely to be female than male; however, viral suppression status did not differ by age or education level. Not surprisingly, the majority of those who were virally suppressed were aware of their infection (86.0%); 14.0% of those suppressed reported being HIV-negative or unknown during the survey. Those who were suppressed were also more likely to have initiated ART (95.3%) than those who were not suppressed (80.3%). Only 33.3% of those not suppressed had evidence of ART analytes, compared to 75.5% of those suppressed.

**Table 1:** Participant characteristics by viral load status, North West Province, South Africa, 2014^a^

	Not Suppressed (≥ 5000 copies/ml) wgt ***n*** = 7981		Suppressed (< 5000 copies/ml) wgt ***n*** = 4976		
	wgt %	95% CI	wgt %	95% CI	***P***-value
Overall	61.6	(51.4–70.9)	38.4	(29.2–48.6)	
Sex					
Male	51.1	(41.0–61.1)	21.1	(10.4–38.2)	0.003
Female	48.9	(38.9–59.1)	78.9	(61.8–89.6)	
Age group					
18–29 Years	22.2	(11.4–38.7)	19.0	(9.2–35.1)	0.868
30–39 Years	45.0	(31.0–59.9)	42.7	(28.1–58.7)	
40–49 Years	32.8	(22.1–45.6)	38.3	(24.5–54.4)	
Education					
Primary or Less	30.2	(20.3–42.4)	35.3	(21.6–52.0)	0.759
Some Secondary	39.3	(30.0–49.5)	39.8	(28.0–53.0)	
Completed Secondary	30.5	(22.2–40.2)	24.8	(13.2–41.8)	
Prior knowledge of HIV serostatus^b^					
Known Positive	54.2	(41.7–66.1)	86.0	(68.6–94.6)	0.005
Unknown	45.8	(33.9–58.3)	14.0	(5.4–31.4)^e^	
ART initiation^b^					
No	19.7	(10.4–34.3)	4.8	(1.4–15.1)	0.040
Yes	80.3	(65.7–89.6)	95.3	(85.0–98.6)	
Time on ART^b^					
< 1 year	23.4	(14.4–35.9)	13.8	(7.4–24.3)	0.134
1–3 years	42.9	(29.4–57.5)	33.7	(18.7–52.8)	
> 3 years	33.7	(21.9–48.0)	52.5	(30.8–58.5)	
Analytes found^c^					
Yes	33.3	(25.3–42.4)	75.5	(57.9–87.4)	< 0.001
No	66.7	(57.6–74.7)	24.5	(12.6–42.2)	
CD4 category (Pima)^d^					
≤ 350 cells/μL	57.8	(42.8–71.5)	28.2	(15.7–45.2)	0.018
> 350 cells/μL	42.2	(28.6–57.2)	71.9	(54.8–84.3)	

^a^Weights account for sampling, non-response, and age/sex of target population

^b^Based on self-report

^c^Assessed for EFV, LPV, NVP

^d^Among those for whom Pima CD4 results were available

^e^Respondents may be mis-reporting status, be elite controllers, or on ART without understanding of their condition [35]

### Resistance patterns

Among the 107 HIV-positive participants with a DBS viral load > 5000 copies/mL and specimens for genotyping, sequences were successfully generated for 71% (*n* = 76), with greater sequencing failures among those who were positive for ART analytes compared to those who were ART-naïve or were negative for ART analytes but with reported prior ART exposure (Fig. 1). Seventy-five (99%) viruses sequenced were pure consensus subtype C using two independent typing tools. One was pure consensus D; none were found to be unique recombinant forms (subtype data not shown).

In weighted analyses, 21.7% of those sequenced had evidence of DRM (Table 2). Resistance was most prominent in those who were initiated on ART with negative analyte results, who likely took ART inconsistently or had interrupted treatment. In this group, 51.1% (95% CI 11.6–89.3%) had any resistance mutation, as compared to 46.6% (95% CI 24.6–70.0%) of those sequenced with positive analyte results and 8.0% (95% CI 3.1–18.9%) among those sequenced who were ART-naïve.

**Table 2:** Resistance by ART Status and drug class, North West Province, South Africa, 2014^a^

	Analyte Positive^**c**^		ART experienced,^**b**^ analyte negative		ART naïve^**b**^		Total	
	Percent	95% CI	Percent	95% CI	Percent	95% CI	Percent	95% CI
**Genotype result**	wgt n^a^ = 2643		wgt n^a^ = 677		wgt n^a^ = 4623		wgt n^a^ = 7943	
No resistance	24.4	14.4–38.3	49.0	10.7–88.4	72.6	55.2–85.1	54.6	43.4–65.4
Any resistance	21.3	9.1–42.4	51.1	11.6–89.3	6.3	2.4–15.5	15.1	8.2–26.3
Genotyping failure	54.3	35.4–72.0	0.0	–	21.1	9.4–40.7	30.3	20.3–42.6
**Resistance - among those genotyped**	wgt n^a^ = 1209		wgt n^a^ = 677		wgt n^a^ = 3650		wgt n^a^ = 5536	
No resistance	53.4	30.0–75.7	49.0	10.7–88.4	92.0	81.0–96.9	78.3	64.3–87.9
Any resistance	46.6	24.6–70.0	51.1	11.6–89.3	8.0	3.1–18.9	21.7	12.1–35.7
**Drug class resistance**	wgt n^a^ = 564		wgt n^a^ = 346		wgt n^a^ = 291		wgt n^a^ = 1201	
Single class								
NRTI only	0.0	–	0.0	–	0.0	–	0.0	–
NNRTI only	0.0	–	73.1	27.9–95.0	63.0	18.1–92.9	36.3	17.7–60.2
PI only	0.0	–	12.2	1.4–58.6	11.5	1.2–58.0	6.3	1.3–25.7
Dual class								
NRTI/NNRTI	100.0	–	14.7	1.6–64.1	0.0	–	51.2	28.3–73.6
PI/NRTI	0.0	–	0.0	–	25.5	3.3–77.6	6.2	0.7–37.3
PI/NNRTI	0.0	–	0.0	–	0.0	–	0.0	–

^a^Weights account for sampling, non-response, and age/sex of target population, ^b^By self report, ^**c**^ Assessed EFV, LPV, NVP

Sequencing results showed a predominance of NNRTI resistance (Table 2). The most common resistance pattern was dual class NRTI/NNRTI resistance at 51.2% (95% CI 28.3–73.6%), followed by single class resistance to NNRTI at 36.3% (95% CI 17.7–60.2%). However, specific mutations differed by presence of analytes. Of the 21 participants successfully sequenced with positive NVP or EFV results, 10 showed two or more DRM, all of whom had both NRTI and NNRTI resistance. The predominant NRTI mutations were M184V (8/10) and K65R (5/10) with three participants harboring both mutations. (Table 3). Among those with NNRTI resistance, the predominant mutations were at codons 100, 101, 103, 106 and 181. In contrast, among participants without evidence of being on ART (analyte negative – regardless of reported ART initiation), 10 harbored at least one major NRTI, NNRTI or PI DRM. Two showed NRTI resistance (M184V) and six harbored NNRTI DRM at codons 101 and 103. The major PI mutations M46I, I50I/V and V82I, which confer low to intermediate resistance to LPV/r used in second line therapy, were detected in three participants. (Table 3).

**Table 3:** Resistance mutations, North West Province, South Africa, 2014

Sex	Age Group	Months on ART	ARV analyte exposure			Viral load	Drug resistance mutation by drug class		
			EFV	NVP	LPV		NRTI	NNRTI	PI
Male	18–29	47	–	–	–	7327	Wild-Type	Wild-Type	V82L
Female	30–39	25	+	+	–	8580	M184V	Y188L	Wild-Type
Male	30–39	N/A	–	–	–	18,800	Wild-Type	Wild-Type	I50V
Female	30–39	21	+	–	–	27,474	K65R, Y115F	V106M, Y181C	Wild-Type
Male	40–49	92	–	–	–	35,300	M184V	K103N, Y188L	Wild-Type
Female	40–49	117	+	–	–	36,873	M41L, M184V, T215F	K101E, V106M, G190A	Wild-Type
Male	30–39	N/A	–	–	–	49,500	M184I	Wild-Type	M46I
Female	30–39	90	+	–	–	53,600	K65R, M184V	K101P, K103S	Wild-Type
Female	30–39	55	+	–	–	56,600	M184V	K103N	Wild-Type
Male	30–39	49	+	–	–	73,200	D67G, T69D, K70R, M184V, K219Q	L100I, K103N	Wild-Type
Female	30–39	N/A	–	–	–	95,059	Wild-Type	K103N	Wild-Type
Female	30–39	47	+	–	–	95,100	K65R, M184V	Y181C, G190S	Wild-Type
Female	30–39	61	+	–	–	120,000	K65R, T69del	K101E, Y181C, G190S	Wild-Type
Male	18–29	N/A	–	–	–	122,000	Wild-Type	K101E	Wild-Type
Male	30–39	22	–	–	–	135,000	Wild-Type	K103N	Wild-Type
Female	30–39	17	–	–	–	145,000	Wild-Type	V106M	Wild-Type
Male	40–49	N/A	–	–	–	390,000	Wild-Type	K103N	Wild-Type
Male	18–29	N/A	+	–	–	415,000	K65R, Y115F, M184V	V106M, G190A	Wild-Type
Male	18–29	15	–	–	–	490,000	Wild-Type	K103N, G190A	Wild-Type
Male	30–39	67	+	–	–	799,000	M41L, D67N, K70E, Y115F, M184V	K103N, V106M	Wild-Type

### Treatment-specific resistance

**Fig. 2:**
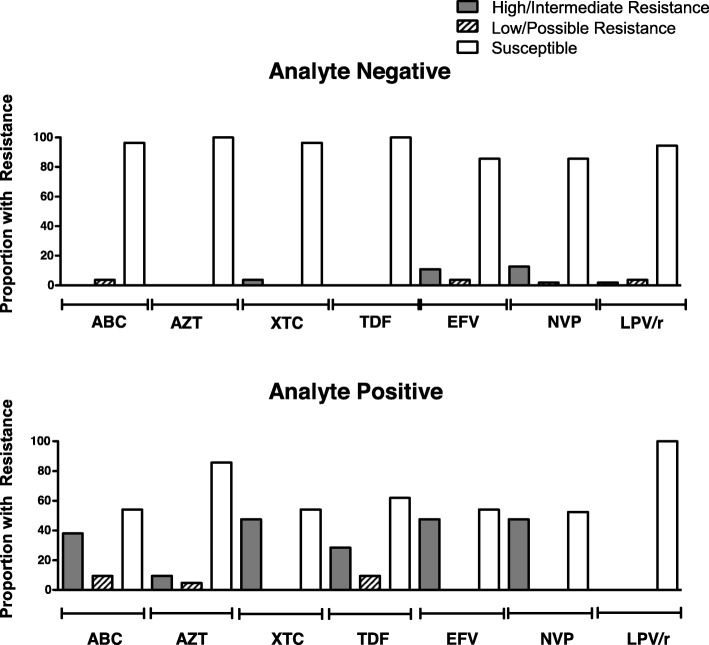
Treatment-specific resistance among analyte negative and analyte positive participants, North West Province, South Africa

There were significant differences in treatment-specific resistance among the analyte positive and negative participants. Among those who were analyte negative, fewer than 5 % demonstrated resistance to azidothymidine (AZT), abacavir (ABC), lamivudine or emtricitabine (XTC), tenofovir disoproxil fumarate (TDF), LPV/r and thus few would fail to suppress using the first-line regimens in South Africa. Among those who were analyte positive, over 10% and as high as 47% showed any resistance to XTC, TDF, EFV, NVP, and ABC and thus are predicted to show reduced susceptibility to first- or second-line regimes containing these antiretrovirals (Fig. 2). Among the participants sequenced, three carried M184V/I with K65R, mutations conferring resistance to the regimen used for oral HIV pre-exposure prophylaxis (PrEP) (FTC/TDF).

## Discussion

We set out to examine the contribution of HIV drug resistance mutations to rates of virologic failure after noting that almost half of HIV-positive participants reporting ART initiation in a population-based sero-prevalence survey had evidence of virologic failure, despite 93% of those in care reporting near perfect adherence [24]. In exploring the factors behind poor viral suppression rates in this rural South African population, drug resistance mutations appear to be making a substantial contribution to sub-optimal outcomes, particularly for those in treatment. Among those sequenced, evidence of DRM was present in 22% of the population and in 47% of those with evidence of ART analytes, with implications for efficacy of treatment regimens.

Little is known about the prevalence of DRM both prior to and following treatment initiation in the general population. One population-based study in South Africa found evidence of DRM in 7% of ART-naïve patients in rural KwaZulu-Natal in 2012, similar to our findings of DRMs in 8% of our 2014 North West population sequenced pre-treatment. Clinical cohort studies in sub-Saharan Africa have found a similar prevalence of drug resistance mutations in 6–11% of ART-naïve patients [11, 20, 22]. If our study participants reported ART initiation accurately, 8% would represent the baseline estimate of transmitted resistance in the population, most of which was NNRTI only (63.0%), with fewer mutations representing PI/NRTI resistance (25.5%) and even fewer being single PI resistance (11.5%). For these patients, DRM could lead to first-line failure. Recent studies have demonstrated that the prevalence of transmitted and acquired drug resistance is increasing in RSA [20]. The cost in a program as large as South Africa’s makes baseline DRM testing prior to ART initiation to inform regimen selection largely infeasible; however NHLS has instituted alert reports for adherence counseling and DRM testing should be made available when treatment failure is evident.

Resistance was particularly high in our sample among those who had initiated ART, and most prevalent among those who had reported initiation but had no evidence of ART analytes, e.g. probable non-adherence. Non-adherence is likely a big contributor to lack of viral suppression and acquired resistance in this sample. It is not possible to distinguish between acquired and transmitted resistance in our data, however, patterns of resistance by ART status provide insights about contributions of acquired and transmitted resistance in this population-based sample. The large majority of DRMs in this population occurred in the ART-initiated population, implying a high likelihood of acquired resistance playing a central role, particularly with all mutations demonstrating NRTI/NNRTI (first-line) dual resistance among the analyte positive group. In fact, research has noted that a high proportion of adults and children who received first line ART will develop virological failure during the first 5 years, with 70–90% of patients in virologic failure acquiring drug-resistant HIV [36]. While this estimate of DRM is higher than that observed in our sample, we were unable to sequence 30% of samples and the majority (22/31) of those were ART positive with viral load measures close to the cut-off (i.e. 20,000 copies or less) for virologic failure. If all ART positive participants with genotyping failure had resistance mutations, this would represent 75.6% of analyte positive participants, coinciding with current estimates [36]. Indeed, if participants were partially suppressed on ART and therefore had positive analytes and lower but not suppressed viral loads, they would be more prone to generate resistance and may lead to an underestimate of DRMs in this population.

The extremely high rates of dual NRTI/NNRTI resistance among the analyte positive group has important implications for the future of the current first-line treatment. Currently the South African national program recommends that providers consider switching patients on the first-line drug regimen if the patient has experienced virological failure (VL > 1000 copies/mL) on at least two occasions two months apart despite good adherence. Our data indicate that approximately one-third of those who report ART intake are negative for analytes, making a premature decision to switch regimens potentially costly and could encourage further DRM. With South Africa currently starting up introduction of a new triple combination regimen of tenofovir disoproxil fumarate, lamivudine (3TC), and dolutegravir (TLD) [37, 38], some progress in stemming resistance to NNRTI could be imminent. However, even with availability of the new regimen, routine monitoring of resistance remains critical [39]. Finally, circulating drug-resistant viruses may have implications for persons initiating PrEP with TDF/FTC. The few seroconversions among individuals enrolled in PrEP trials occurred among those who started PrEP during unrecognized acute HIV infection, and the emergence of resistance primarily involved M184V/I, which can be selected for under the selective pressure of FTC [40–42].

A strength of this study is the population-based sample in an understudied area with data on viral load, ART analytes, and DRMs. Our data also has several limitations. Only 70% of the samples were sequenced, with failure being highest for those not suppressed on ART and with comparatively lower viral load measures, which could lead to an underestimation of DRMs if that population is intermittently adherent. Studies restricted to ART-naïve populations using the same medium for genotyping have sequencing success of closer to 92% [43]. Additionally, ART initiation and adherence are based on self-report; however, the use of analyte data as a biological marker of initiation and adherence improves the study’s accuracy. We cannot distinguish between acquired and transmitted resistance in our data, and, finally, we used DBS instead of plasma for the assays. Though DBS offer a feasible, low-resource alternative for viral load and DRM surveillance, they have well documented limitations – notably cellular HIV DNA contributes to copy number when using whole blood instead of blood plasma samples [29]. We therefore used a conservative threshold of 5000 copies/mL for defining viral suppression, and, as a result, run the risk of over-estimating viral suppression. Even so, viral suppression in this study aligns well with data from the NHLS at the time of the study, which suggested that viral suppression among PLHIV in care (defined as < 400 copies/mL plasma) ranged from 52 to 75% for North West Province, depending on the district [44], similar to our estimates among those on treatment.

## Conclusion

We found that both non-adherence and drug resistance mutations likely play a key role in virologic failure in this rural community in North West Province, South Africa. Increasing ART coverage can significantly lower the risk of new HIV infections in South Africa [45]; however, gains in treatment expansion will be lost if inconsistent adherence is not addressed and resistance continues to increase. There is growing evidence that pre-treatment drug resistance is increasing in sub-Saharan Africa [22, 23], making it essential that policies around treatment regimens are reviewed and updated frequently, and that surveillance be instituted in both HIV-positive, ART-naïve populations and among those failing treatment, including monitoring of drug exposure for non-adherence and treatment failure despite adherence. Countries should consider instituting sentinel sites for baseline drug resistance testing in settings where feasible.

## Data Availability

The datasets generated and analysed during the current study are not publicly available to ensure confidentiality of participants’ private health information. De-identified, aggregate data will be made available from the corresponding author upon reasonable request and with IRB approval.

## Abbreviations

ABC: Abacavir
ART: Antiretroviral therapy
AZT: Azidothymidine
DBS: Dried blood spots
DRM: HIV drug resistance mutations
DU: Dwelling unit
EA: Enumeration area
EFV: Efavirenz
LC MS/MS: Liquid chromatography-tandem mass spectrometry method
LPV: Lopinavir
MRM: Multiple reaction monitoring
NNRTI: Non-nucleoside reverse transcriptase inhibitors
NRTI: Nucleoside reverse transcriptase inhibitors
NVP: Nevirapine
PCR: Polymerase chain reaction
PI: Protease inhibitors
PrEP: HIV pre-exposure prophylaxis
Pro: Protease
RSM: Dr. Ruth Segomotsi Mompati District
RT: Reverse transcriptase
StatsSA: Statistics South Africa
TDF: Tenofovir disoproxil fumarate
TLD: Dolutegravir
UNAIDS: Joint United Nations Programme on HIV/AIDS
XTC: Lamivudine or emtricitabine
